# Characterization of a Novel Cysteine Protease Inhibitor from Poultry Red Mites: Potential Vaccine for Chickens

**DOI:** 10.3390/vaccines9121472

**Published:** 2021-12-13

**Authors:** Sotaro Fujisawa, Shiro Murata, Masayoshi Isezaki, Takuma Ariizumi, Takumi Sato, Eiji Oishi, Akira Taneno, Naoya Maekawa, Tomohiro Okagawa, Osamu Ichii, Satoru Konnai, Kazuhiko Ohashi

**Affiliations:** 1 Department of Disease Control, Faculty of Veterinary Medicine, Hokkaido University, Kita 18, Nishi 9, Kita-ku, Sapporo 060-0818, Japan; s.fujisawa@vetmed.hokudai.ac.jp (S.F.); m-isezak@photon.chitose.ac.jp (M.I.); ariizumi@czc.hokudai.ac.jp (T.A.); konnai@vetmed.hokudai.ac.jp (S.K.); okazu@vetmed.hokudai.ac.jp (K.O.); 2 Department of Advanced Pharmaceutics, Faculty of Veterinary Medicine, Hokkaido University, Sapporo 060-0818, Japan; maekawa@vetmed.hokudai.ac.jp (N.M.); okagawa@vetmed.hokudai.ac.jp (T.O.); 3 Division of Molecular Pathology, International Institute of Zoonosis Control, Hokkaido University, Sapporo 001-0020, Japan; 4 Vaxxinova Japan K.K., Tokyo 105-0013, Japan; t-sato@vaxxinova.co.jp (T.S.); e-oishi@vaxxinova.co.jp (E.O.); a-taneno@vaxxinova.co.jp (A.T.); 5 Department of Basic Veterinary Science, Faculty of Veterinary Medicine, Hokkaido University, Sapporo 060-0818, Japan; ichi-o@vetmed.hokudai.ac.jp; 6 Laboratory of Agrobiomedical Science, Faculty of Agriculture, Hokkaido University, Sapporo 060-8589, Japan

**Keywords:** *Dermanyssus gallinae*, poultry red mite, cystatin, vaccine, cocktail vaccine

## Abstract

Poultry red mite (PRM; *Dermanyssus gallinae*) is a hazardous, blood-sucking ectoparasite of birds that constitutes a threat to poultry farming worldwide. Acaricides, commonly used in poultry farms to prevent PRMs, are not effective because of the rapid emergence of acaricide-resistant PRMs. However, vaccination may be a promising strategy to control PRM. We identified a novel cystatin-like molecule in PRMs: *Dg-Cys*. *Dg-Cys* mRNA expression was detected in the midgut and ovaries, in all stages of life. The PRM nymphs that were artificially fed with the plasma from chickens that were immunized with *Dg-Cys* in vitro had a significantly reduced reproductive capacity and survival rate. Moreover, combination of *Dg-Cys* with other antigen candidates, like copper transporter 1 or adipocyte plasma membrane-associated protein, enhanced vaccine efficacies. vaccination and its application as an antigen for cocktail vaccines could be an effective strategy to reduce the damage caused by PRMs in poultry farming.

## 1. Introduction

Poultry red mites (*Dermanyssus gallinae*, PRM) are blood-sucking ectoparasites that infest chickens. Mass infestation by PRMs negatively affects chickens in various ways, causing such conditions as anemia and depression, resulting in significant losses of the productivity in poultry farming. Since continuous use of chemical acaricides, which are commonly used to prevent PRM infestations, could cause various problems such as the selection of drug-resistant PRMs and the contamination of products with acaricides, an alternative control method is needed. Over the past few years, vaccination has been in the spotlight as a novel strategy for the control of ectoparasites, including PRMs, and several studies have examined antigen candidates for their efficacies [1,2,3,4,5]. However, adequate anti-PRM efficacy has not been reported through practical applications in the field [6]. Thus, vaccination strategies need to be improved, while more effective vaccine antigens need to be explored to increase vaccine efficacy. As for the target molecules of vaccines against ectoparasites, two types of proteins may be considered: proteins secreted into the parasite’s saliva that facilitate attachment to the host and blood sucking by mitigating host immune responses, and proteins expressed in the parasite’s midgut, which is the middle part of the digestive tract. Since the midgut is the reservoir for ingested blood, the latter proteins could be frequently and efficiently exposed to the antibodies found in the blood obtained from immunized animals. For blood feeding, tick species infest hosts for long periods (for three to ten days or more [7]). Meanwhile, PRMs infest chickens within a few minutes to one hour and do not stay on the chicken’s body after blood sucking. In addition, PRMs are intermittent feeders that repetitively suck blood in their life cycle [8]. Therefore, midgut proteins may be more appropriate as vaccine antigens against PRMs than salivary gland proteins.

Cystatins are reversible inhibitors of papain-like cysteine proteases and legumains [9] and regulate various biological processes, including innate immunity, epidermal homeostasis, and apoptosis in vertebrates [10,11,12]. Similarly, cystatins play crucial roles in the lives of parasites, and bmcystatin isolated from *Rhipicephalus microplus* is involved in embryogenesis, as it inhibits vitelline-degrading peptidase [13]. In addition, cystatins found in *Haemaphysalis longicornis* and *Ornithodoros moubata* regulate blood digestion, heme detoxification, and tick innate immunity [14,15,16]. The knockdown of cystatins by gene silencing, or the blockade of cystatin activities using specific antibodies, significantly reduces the efficiency of blood feeding in *Amblyomma Americanum* [17] and increases mortality rates in *Ornithodoros moubata* [18]. Collectively, cystatins are indispensable for hematophagous ectoparasites; hence, these molecules could be effective antigens for vaccines.

Vaccine antigen targets should be highly and constitutively expressed or at least be expressed in blood-fed PRMs. To investigate such molecules, we previously conducted a comparative analysis of the transcriptome of blood-fed and starved PRMs [19] and identified a novel cystatin-like transcript, *Dg-Cys*, that was highly expressed in both blood-fed and starved states. In this study, we characterized the full-length sequence of *Dg-Cys* and evaluated the physiological functions of *Dg-Cys* along with its potential as a vaccine antigen. In addition, we assessed the acaricidal effects of mixed plasmas containing antibodies against multiple antigens (including *Dg-Cys*) to evaluate the anti-PRM properties of the “cocktail vaccine”.

## 2. Materials and Methods

### 2.1. Ethics Approval and Consent of Participants

All animal experiments were approved by the Institutional Animal Care and Use Committee, Hokkaido University (Approval number: 20–0051). Moreover, all experiments were performed in accordance with relevant guidelines and regulations of the Faculty of Veterinary Medicine, Hokkaido University, which has been enacted confirming to the ARRIVE guidelines and fully accredited by the Association for Assessment and Accreditation of Laboratory Animal Care International (AAALAC).

### 2.2. PRM Samples

PRM samples were prepared as described previously [4,5,19]. PRMs of mixed developmental stages and sexes were obtained from an egg-laying farm in Japan. PRMs were stored in a TubeSpin Bioreactor 600 (TPP Techno Plastic Products AG, Trasadingen, Switzerland). A part of the dark red, round PRMs was designated as “blood-fed PRMs” and collected using 1200 μL extra-long filter tips (WATSON Bio Lab, Tokyo, Japan) within 2 days of sample collection. The remaining PRMs were maintained at 25 °C in 70% humidity for a 2-week period and designated as “starved PRMs.” Some of the blood-fed and starved PRMs were fixed with 70% ethanol, and then eggs, larvae, protonymphs, deutonymphs, and adults were segregated through microscopic observation. The residual PRMs were stored at −80 °C until use.

### 2.3. RNA Isolation and cDNA Synthesis

RNA isolation and cDNA synthesis from PRM samples were performed as described previously [4,5]. Each collected PRM sample was suspended in 600 μL of Buffer RLT Plus (RNeasy Plus Mini Kit) (Qiagen, Hilden, Germany) and homogenized thoroughly using a 1.5-mL Homogenization Pestle for a 1.5-mL microcentrifuge tube (Scientific Specialties, Inc., Lodi, CA, USA). Total RNA was isolated using the RNeasy Plus Mini Kit according to the manufacturer’s instructions. cDNA was synthesized from the isolated RNA using PrimeScript Reverse Transcriptase (Takara Bio Inc., Shiga, Japan) using 200 pmol of oligo (dT) 18 primer (Hokkaido System Science, Hokkaido, Japan).

### 2.4. Identification of the Full-Length Nucleotide Sequence of Dg-Cys in PRMs

A novel cystatin-like transcript, *Dg-Cys*, was previously identified by RNA-Seq analysis [19] (BioSample accession number: SAMD00228960, SAMD00229086). For identifying the full-length nucleotide sequence of the cDNA encoding *Dg-Cys* transcript, rapid amplification of cDNA ends (RACE) was performed using total RNA isolated from blood-fed PRMs and 5′ and 3′ RACE systems (Invitrogen, Carlsbad, CA, USA) according to the manufacturer’s instructions. The following primers were designed and used: For 5′ RACE: GSP1, 5′-ACC GAT CAC TAC AAA CTG CA-3′; GSP2, 5′-CAA CAG CAG CAG CAG TAC GA-3′; GSP3, 5′-AGT ACG AAT AAA CAC GCG CG-3′. For 3 RACE: GSP1, 5′-CCA AAC GGT CTC CGT ACT GT-3′; GSP2, 5′-CCG TAC TGT CGG ACG AAA TC-3′. The signal peptide domain and the cystatin-conserved site were predicted using the SignalP software (http://www.cbs.dtu.dk/services/SignalP/, accessed on 11 August 2019) and InterProScan program v5.32-71.0 (https://www.ebi.ac.uk/interpro/search/sequence/, accessed on 24 September 2019), respectively. The phylogenic tree was constructed by MEGA software version X (https://www.megasoftware.net/show_eua, accessed on 3 October 2018 [20]), using a maximum likelihood of 1000 bootstrap replicates and a JTT matrix-based model [21], by using a discrete Gamma distribution (+G) and assuming that a certain fraction of sites is evolutionarily invariable (+I), to improve the tree topology.

### 2.5. Laser-Capture Microdissection

cDNA synthesis from salivary glands, midguts, and ovaries was performed using laser-capture microdissection (LCM), as previously described [22]. Briefly, starved PRMs of mixed developmental stages were fixed in carnoy solution, embedded in paraffin, and cut into 5-μm thick sections. Sections were mounted on glass slides precoated with LCM films (Meiwafosis, Tokyo, Japan) and stained with 1% toluidine blue, and each tissue was dissected using MicroBeam Rel.4.2 (Carl Zeiss, Oberkochen, Germany). Total RNA was extracted from the tissues using the RNAqueous^®^-Micro Kit (Thermo Fisher Scientific, Waltham, MA, USA) according to the manufacturer’s protocol, and cDNA synthesis was performed using the SuperScript^TM^ First-Strand Synthesis System for RT-PCR (Invitrogen, Thermo Fisher Scientific, 168 Third Avenue, Waltham, MA, USA 02451), using 300 pmol of random hexamer primer (Hokkaido System Science).

### 2.6. Gene Expression Analysis

The expression of *Dg-Cys* in each feeding state, tissue, and developmental stage was examined by RT-PCR with Ex-Taq polymerase (Takara Bio Inc.), using the synthesized cDNA, as described above. The specific primer sets used were as follows: *Dg-Cys*-for, 5′-GTC TTT GCC TTC CAG TCG AG-3′; *Dg-Cys*-rev, 5′-GGT CTA GCT TGC TCC AAA CG-3′. As an internal control, *elongation factor 1-alpha 1*-like (*Elf1a1*) was amplified using the following primer set [23]: Elf1a1-for, 5′-GTC GGT GTC ATC AAG TCC GT-3′; Elf1a1-rev, 5′-AGG GTC GAG AGT GTA GGG TC-3′. Since the RNA samples extracted from salivary glands were small in quantity or of low quality, the detection of target genes in cDNA samples synthesized from salivary glands was performed by nested PCR using the same primer set.

### 2.7. Real-Time Quantitative RT-PCR

To quantify the expression of *Dg-Cys* mRNA at each developmental stage and feeding state of PRMs, quantitative PCR was performed on cDNA samples from different life stages, except for eggs, and for different feeding states using LightCycler480^®^ System II (Roche Diagnostics, Mannheim, Germany) and TB Green Premix DimerEraser (Takara Bio Inc.) according to the manufacturers’ instructions. To amplify the *Dg-Cys* gene, the primer set *Dg-Cys*-for and *Dg-Cys*-rev was used, while the primer set Elf1a1-for and Elf1a1-rev was used to amplify *Elf1a1* as an internal control. The cycling conditions included an initial denaturation step at 95 °C for 30 s, followed by 45 cycles of 95 °C for 5 s, 60 °C for 30 s, and 72 °C for 30 s. To evaluate the specificities of primer pairs, a final melting curve analysis was performed from 65 °C to 95 °C at a rate of 0.1 °C/s. To generate standard curves for quantification, serial dilutions of T-vector pMD20 (Takara Bio Inc.) containing *Dg-Cys* or *Elf1a1* were used. Each sample was tested four times, and *Dg-Cys* mRNA expression was calculated as a ratio by dividing the concentration of *Dg-Cys* mRNA by that of *Elf1a1* mRNA.

### 2.8. Preparation of Recombinant Dg-Cys

Recombinant *Dg-Cys* was expressed as a fusion protein with a histidine tag (*Dg-Cys*-his), using the BIC system (Takara Bio Inc.). Specific primers containing homologous recombination sites were designed to express *Dg-Cys*-his, according to the manufacturer’s instructions: pBIC4-*Dg-Cys*-for, 5′-GAT GAC GAT GAC AAA GGC CTT TCG GAC GTG GCC G-3′; pBIC4-*Dg-Cys*-rev, 5′-CAT CCT GTT AAG CTT TTA ATT CTC GCA CCG CTT C-3′. PCR was performed using KOD-Plus-Neo (TOYOBO Co., Ltd., Osaka, Japan) to amplify the open reading frame (ORF) of *Dg-Cys* lacking the signal peptide region, and the fragments were integrated into the cloning site of the pBIC4 vector (Takara Bio Inc.) and transformed into *Brevibacillus* competent cells by homologous recombination. The transformed bacteria were cultured in 2SY medium for 48 h at 32 °C, as per the manufacturer’s instructions. The supernatants were then collected, and proteins were purified using Ni Sepharose 6 Fast Flow (Thermo Fisher Scientific). The buffer was replaced with phosphate-buffered saline (PBS) using SnakeSkin^TM^ Dialysis Tubing, 10K MWCO (Thermo Fisher Scientific) overnight at 4 °C. To confirm protein purification, the obtained proteins were mixed with 2× SDS buffer (125 mM Tris–HCl pH 6.8, 4% SDS, 10% 2-mercaptoethanol, and 20% glycerol), boiled for 5 min, separated using 14% SDS-polyacrylamide gel electrophoresis (SDS-PAGE), and stained with Coomassie brilliant blue (CBB, FUJIFILM Wako Pure Chemical Corporation, Osaka, Japan). The protein concentration was determined using the Pierce^TM^ BCA Protein Assay Kit (GE Healthcare, Chicago, IL, USA), according to the manufacturer’s instructions.

### 2.9. Enzymatic Activity of Dg-Cys-his

The inhibitory properties of *Dg-Cys*-his against cathepsin L, S, and B were assessed using the SensoLyte Rh110 Cathepsin L Assay Kit (ANASPEC, Fremont, CA, USA), SensoLyte 520 Cathepsin S Assay Kit (ANASPEC), and SensoLyte 520 Cathepsin B Assay Kit (ANASPEC), respectively, according to the manufacturer’s instructions. Bovine serum albumin (Sigma-Aldrich, St. Louis, MO, USA) (BSA) diluted in PBS was used as a negative control. Y-axis indicates the percentage of enzymatic activities of cathepsin L, S, and B in the presence of *Dg-Cys*-his compared to that in the presence of BSA.

### 2.10. Immunization of Chickens with Dg-Cys-his

Plasma was extracted from chickens immunized with *Dg-Cys*-his (Supplementary Figure S1). Specifically, purified *Dg-Cys*-his was mixed with light liquid paraffin as an adjuvant (20 μg/mL). An emulsion of PBS and light liquid paraffin was prepared and used as a control. Four chickens (Hy-Line Brown) were subcutaneously immunized with 20 μg of *Dg-Cys*-his at 3 weeks of age. Four weeks later, chickens in the immunized group were immunized again with 20 μg of *Dg-Cys*-his in light liquid paraffin. Heparinized blood was collected 3 weeks after the second immunization, and the plasma was isolated by centrifugation at 2000× *g* for 10 min. As a control, two chickens were subcutaneously immunized with PBS, and plasma was isolated 3 weeks after the second immunization.

### 2.11. Enzyme-Linked Immunosorbent Assay (ELISA)

Antibody titers in plasma samples were determined by ELISA. The purified *Dg-Cys*-his was coated onto the wells of 96-well plates (Sumitomo Bakelite Co., Ltd., Tokyo, Japan) (100 ng/well) for 16 h in a carbon-bicarbonate buffer. After washing each well three times with PBS, PBS containing 0.05% Tween 20 (PBS-T) and 1% BSA was added and incubated at 37 °C for 2 h. The wells were then washed five times with PBS-T, and plasma samples diluted at 2000×, 4000× 8000×, and 16,000× with PBS were added. After incubation for 1 h at 25 °C, the wells were washed five times with PBS-T and incubated with anti-chicken IgY peroxidase rabbit antibodies (Sigma-Aldrich, A9046) diluted at 5000× with PBS-T for 1 h at 25 °C. Finally, the wells were washed five times with PBS-T and allowed to react with the TMB one-component substrate (Bethyl Laboratories, Montgomery, TX, USA) for 20 min at 25 °C, in the dark. The reaction was quenched with 0.18 M H_2_SO_4_, and the absorbance was measured at 450 nm. The assay was performed in duplicate.

### 2.12. Western Blotting

The production of specific antibodies against *Dg-Cys*-his was examined by Western blotting, as described previously [4,5]. Purified *Dg-Cys*-his was separated using a 14% SDS-polyacrylamide gel and then transferred to polyvinylidene difluoride membranes (Merck Millipore, Burlington, MA, USA). The membrane was blocked overnight at 4 °C with PBS-T containing 1% skim milk. The membranes were incubated at 25 °C with the isolated plasmas from the immunized chickens, washed three times with PBS-T, and incubated at 25 °C with anti-chicken IgY peroxidase rabbit antibodies (Sigma-Aldrich, A9046) diluted at 5000×. Finally, the membranes were incubated with Immobilon Western Chemiluminescent HRP Substrate (Merck Millipore) to visualize the peroxidase reaction. To analyze the specificities of antibodies, the His-tagged recombinant protein of *D. gallinae* copper transporter 1 (Dg-Ctr1-N-his) was also incubated with plasma from the *Dg-Cys*-his-immunized chickens [4] in the same manner as described above. To visualize Dg-Ctr1-N-his, SDS-PAGE and CBB staining was also performed as described above.

### 2.13. Evaluation of the Potential of Dg-Cys as a Vaccine Antigen

The potential of *Dg-Cys* as a vaccine antigen was analyzed through an in vitro feeding assay [23]. Fresh chicken blood was collected from healthy chickens maintained at the Field Science Center for Northern Biosphere, Hokkaido University, and incubated at 40 °C before use. After centrifugation at 2000× *g* for 10 min, the plasma was replaced with an equal volume of immune plasma as described above. Approximately 100 PRMs of mixed developmental stages were collected in the artificial feeding devices as previously described [23], and the devices were capped with rubber caps type 2 (GE Healthcare). For ventilation, the rubber cap was penetrated using a 27-G needle (TERUMO CORPORATION, Tokyo, Japan). The top of the devices were filled with 400 µL of blood, and blood feeding was performed for 4 h at 40 °C in a dark, humid environment with modest shaking. Only blood-fed PRMs were collected using Pasteur pipettes (day 0) and kept at 25 °C in 70% humidity during the observation period. The number of dead PRMs was monitored for 1 week, and the numbers of newborn larvae, protonymphs, and cast-off skins were counted at day 7. The anti-PRM property of *Dg-Cys*-his immunization was evaluated based on the following assessment items:

(1) The SR:

$$\mathrm{SR}\;\left(\%\right)=\left(1-\frac{\mathrm{No}.\;\mathrm{of}\;\mathrm{dead}\;\mathrm{PRMs}\;\mathrm{on}\;\mathrm{day}\;7}{\mathrm{No}.\;\mathrm{of}\;\mathrm{blood}-\mathrm{fed}\;\mathrm{PRMs}}\right)\times 100$$

(2) The RC:

$$\mathrm{RC}\;=\frac{\mathrm{No}.\;\mathrm{of}\;\mathrm{new}\;\mathrm{borne}\;\mathrm{larvae}\;\mathrm{and}\;\mathrm{protonymphs}\;\mathrm{on}\;\mathrm{day}\;7}{\mathrm{No}.\;\mathrm{of}\;\mathrm{blood}-\mathrm{fed}\;\mathrm{adults}}$$

(3) The molting rate (MR):

$$\mathrm{MR}\;\left(\%\right)=\frac{\mathrm{No}.\;\mathrm{of}\;\mathrm{cast}-\mathrm{off}\;\mathrm{skins}\;\mathrm{on}\;\mathrm{day}\;7}{\mathrm{No}.\;\mathrm{of}\;\mathrm{blood}-\mathrm{fed}\;\mathrm{protonymphs}\;\mathrm{and}\;\mathrm{deutonymphs}}\times 100$$

The feeding assay was performed twice. In the first experiment, the SR and RC were assessed; to measure the RC accurately, deutonymphs were excluded from the analysis. In the second experiment, the SR and MR were evaluated. To precisely evaluate the MR, adults were not included in the analysis in the second experiment. The SR, RC, and MR were analyzed by comparing the total number of PRMs or cast-off skins in the immunized and control groups.

### 2.14. Evaluation of the Efficacy of the “Cocktail Vaccine”

To assess the effects of the “cocktail vaccine”, plasmas from chickens immunized with copper transporter 1-like molecule (Dg-Ctr1 [4]) or adipocyte plasma membrane-associated protein-like molecule (Dg-APMAP [5]) were mixed with an equal volume of immune plasmas of *Dg-Cys*. As a control, equal volumes of plasmas from chickens immunized with PBS, as described above, were mixed with plasmas prepared in our previous studies [4,5]. Blood feeding was conducted as described above using adult PRMs, and the SR was monitored for a 10-day period.

### 2.15. Statistics

For gene expression analyses, differences were analyzed using the Mann–Whitney *U* test. For in vitro feeding assay, the SR, RC, and MR between the *Dg-Cys*- and PBS-immunized groups were compared by Fisher’s exact test. The odds ratio and 95% confidential interval (CI) was estimated. In addition to assessing the difference in the SR, Kaplan–Meier curves were generated and a log-rank test was conducted. For multiple comparisons, Fisher’s multicomparison test and a *p* value was adjusted by Holm method. *p* values of <0.05 and <0.01 were considered statistically significant.

## 3. Results

### 3.1. Cloning and Sequence Analysis of Dg-Cys from PRMs

**Figure 1:**
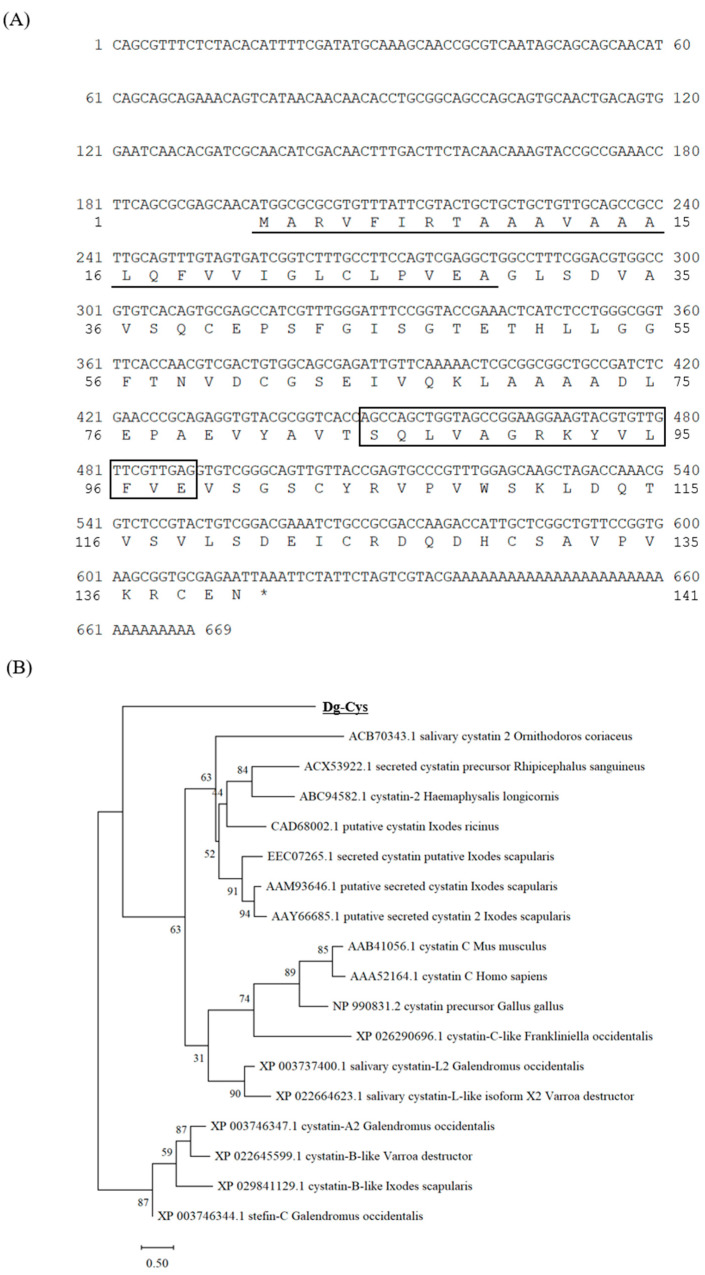
Cloning of a cystatin-like molecule from *Dermanyssus gallinae*. (**A**) Nucleotide and deduced amino acid sequences of cDNA encoding a *D. gallinae* cystatin-like molecule (*Dg-Cys*). Putative signal peptide sites were underlined, and the cystatin-conserved site was boxed. (**B**) A phylogenic tree based on the deduced amino acid sequence of *Dg-Cys*. The tree was built with the maximum likelihood method using the MEGA X software [20]. Numbers indicate bootstrap percentage (1000 replicates). The scale indicates the divergence time. *Stop codon.

We obtained the transcript of the cystatin-like molecule, which showed high expression levels in both blood-fed and starved PRMs, from the dataset of our RNA-Seq analysis [19] and designated the transcript as *Dg-Cys*. We did not observe any significant difference in the expression of *Dg-Cys* between the two feeding states of PRMs (Supplementary Table S1). We performed 5′ and 3′ RACE analyses to reveal that the full-length cDNA was 669 bp, and the ORF was 423 bp, encoding a 140 amino-acid protein. The deduced amino acid sequence of *Dg-Cys* contained a putative signal peptide in positions 1–29 and a cystatin-conserved site at positions 85–98 (Figure 1A). Phylogenetic analysis using cystatins from chickens, mammals, ticks, and mites revealed that *Dg-Cys* formed a distinct cluster, but was closer to secreted cystatins (cystatin 2, cystatin C) rather than intracellular ones (stefin, cystatins A and B) (Figure 1B).

### 3.2. Dg-Cys Expression Profile

**Figure 2:**
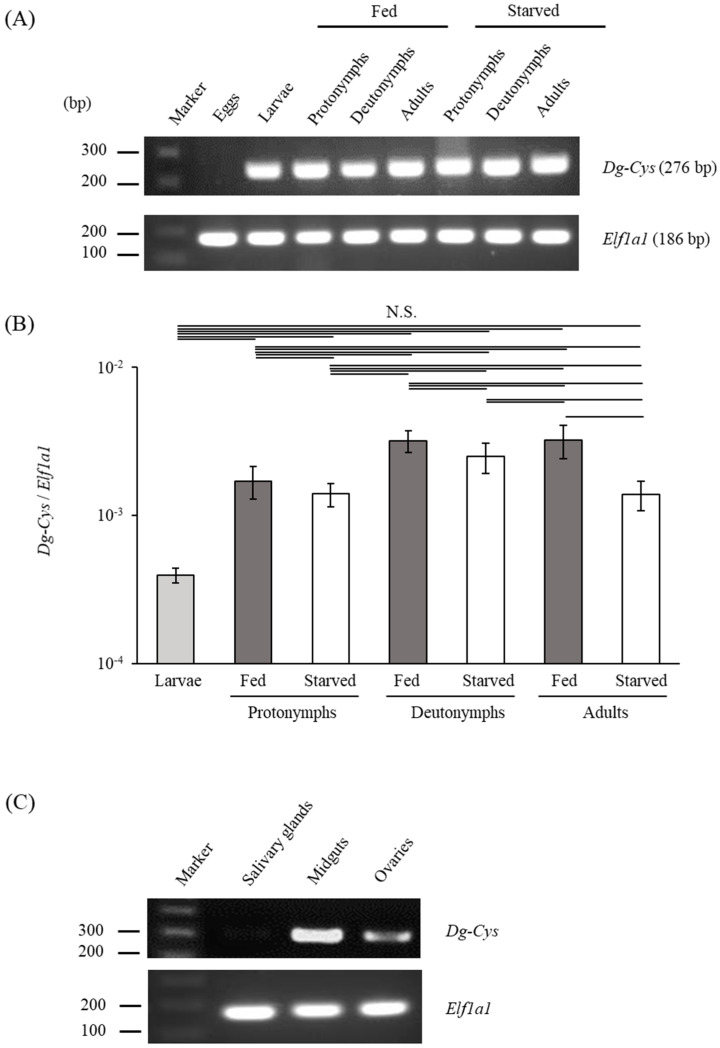
Gene expression analysis of *Dg-Cys*. *Dg-Cys* expression was examined by RT-PCR/nested PCR at each life-stage and blood-feeding state (**A**), and in different tissues of PRMs (**C**). *Elongation factor 1-alpha 1*-like gene (*Ef1a1*) was amplified as an internal control. (**B**) Real-time quantitative RT-PCR was performed to quantify the gene expression of *Dg-Cys* at each life-stage and blood-feeding state of PRMs. The extent of *Dg-Cys* expression was calculated by dividing the copy numbers of *Dg-Cys* by those of *Elf1a1*. Each experiment was repeated four times and error bars indicate SEM. Statistical analyses were performed using Steel–Dwass test. N.S.: not significant. (**C**) The expression of *Dg-Cys* in the midguts and ovaries was analyzed by RT-PCR, and the expression in the salivary glands was analyzed by RT-nested PCR.

To investigate the gene expression profiles of *Dg-Cys*, we first analyzed the expression of *Dg-Cys* mRNA at different life-stages. Real-time reverse transcription polymerase chain reaction (RT-PCR) and real-time quantitative RT-PCR analyses revealed that *Dg-Cys* mRNA was expressed in all life stages, except in eggs, and was detected regardless of the feeding state (Figure 2A,B and Figure S3). Moreover, the expression analysis of *Dg-Cys* in the midgut, salivary glands, and ovaries by LCM and RT-PCR/nested PCR showed that *Dg-Cys* was clearly expressed in the midgut and ovaries, whereas *Dg-Cys* was not detectable in the salivary glands (Figure 2C and Figure S3).

### 3.3. Dg-Cys Enzymatic Activity

**Figure 3:**
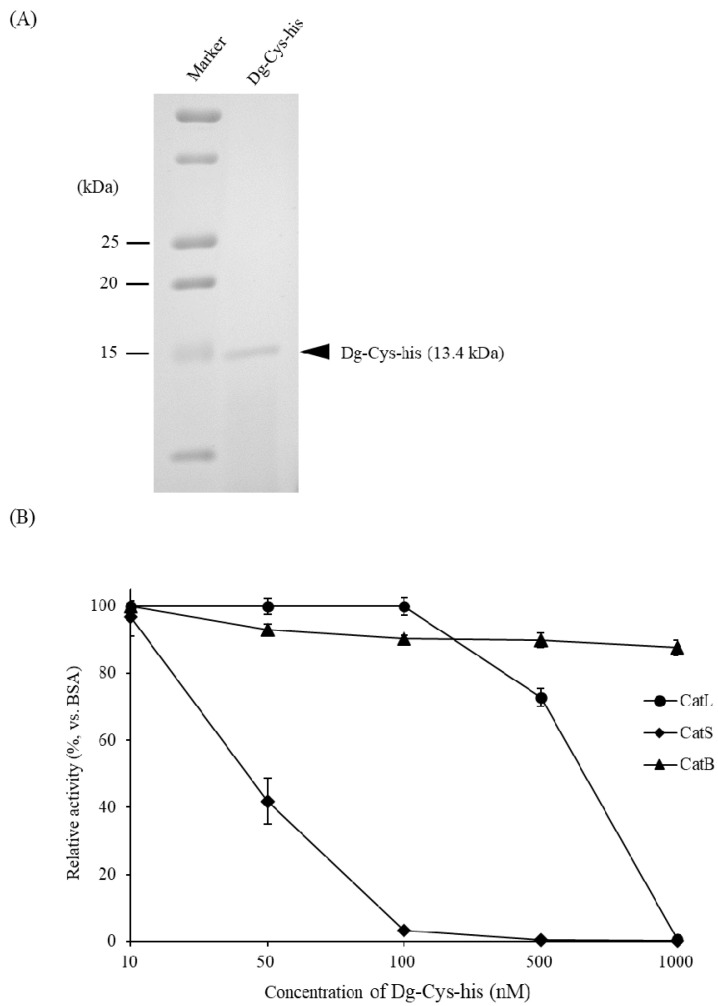
Functional analysis of recombinant *Dg-Cys*. (**A**) The recombinant protein of *Dg-Cys* (*Dg-Cys*-his) was prepared using BIC system. Purified *Dg-Cys*-his was separated by SDS-PAGE and visualized by staining with Coomassie brilliant blue. (**B**) Inhibitory properties of *Dg-Cys*-his against cysteine proteases were examined. Cathepsins L, S, and B were incubated with their fluorometric substrates in the presence of *Dg-Cys*-his or bovine serum albumin (BSA). The X-axis indicates the concentrations of *Dg-Cys*-his. The Y-axis indicates relative enzymatic activities of each cysteine proteases in the presence of *Dg-Cys*-his compared to those in the presence of BSA. The assays were performed in triplicate, and error bars indicate SEM.

By using the BIC system, we prepared the recombinant *Dg-Cys* as a fusion protein with a His-tag (*Dg-Cys*-his). *Dg-Cys*-his was purified from the culture supernatant of the transformed bacteria, and the expression and purity of *Dg-Cys*-his were confirmed by sodium dodecyl-sulfate polyacrylamide gel electrophoresis (SDS-PAGE) and Coomassie brilliant blue staining. *Dg-Cys*-his was detected at the predicted molecular weight (approximately 13.4 kDa) (Figure 3A and Figure S4). Next, we investigated the function of *Dg-Cys* protein. Specifically, we assessed the inhibitory properties of *Dg-Cys*-his against cysteine proteases using commercial kits to measure the enzyme activities of cysteine proteases, cathepsins L, B, and S. The enzymatic activity of cathepsins L and S was suppressed in the presence of *Dg-Cys*-his in a dose-dependent manner (50% inhibitory concentration for cathepsins L and S: 666.8 nM and 36.1 nM, respectively), whereas that of cathepsin B was not inhibited (Figure 3B). These data suggest that *Dg-Cys*-his functions as an inhibitor of cysteine proteases.

### 3.4. Acaricidal Potential of the Plasma from Chickens Immunized with Dg-Cys-his

**Figure 4:**
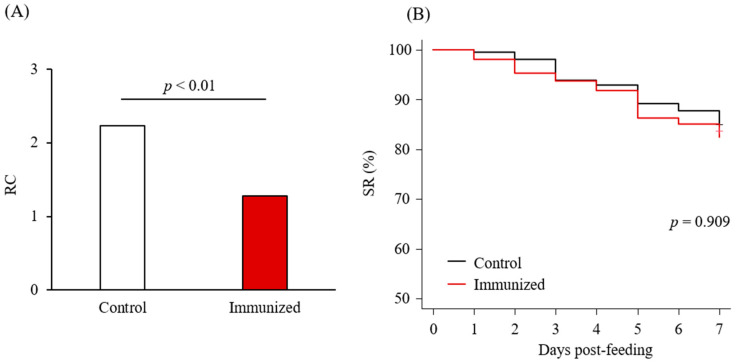
Anti-PRM effects of plasma from chickens immunized with *Dg-Cys*-his (first experiment). (**A**) The reproductive capacity (RC) at seven days post-feeding was assessed. Statistical analysis was performed using Fisher’s exact test. *p* < 0.01 was considered statistically significant. (**B**) The survival rate (SR) of PRMs that were fed with the plasma from immunized chickens was assessed every day for a one-week period. Statistical analysis was performed using Log-rank test.

**Figure 5:**
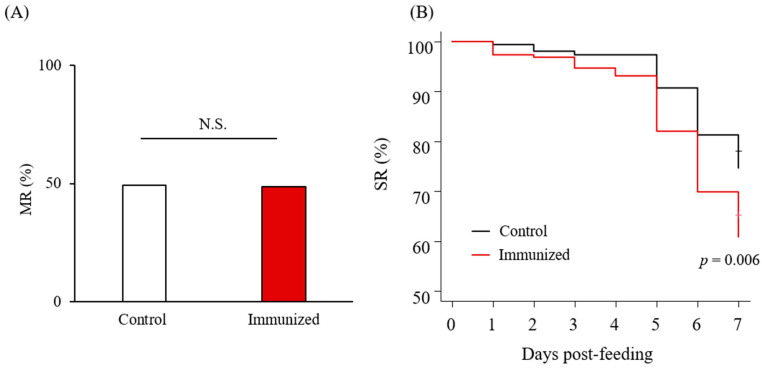
Anti-PRM effects of plasma from chickens immunized with *Dg-Cys*-his (second experiment). (**A**) The molting rate (MR) at seven days post-feeding was assessed. Statistical analyses were performed using Fisher’s exact test. (**B**) The survival rate (SR) of PRMs that were fed with the plasma from immunized chickens was assessed every day for a one-week period. Statistical analyses were performed using Log-rank test. *p* < 0.01 was considered statistically significant. N.S.: not significant.

Chickens were subcutaneously immunized twice with *Dg-Cys*-his as an emulsion with light liquid paraffin before collecting their plasma (Supplementary Figure S1). Antibody titers in the plasma samples were determined by ELISA, and two of the immunized chickens, IM2 and IM4, showed higher antibody titers against *Dg-Cys*-his (Supplementary Table S2). Further Western blot analysis revealed that the antibodies produced in IM2 and IM4 contained antibodies specific to *Dg-Cys*-his (Supplementary Figures S2 and S5). Plasma from IM2 and IM4, and from two chickens of the control group, were subjected to an in vitro feeding assay [23] to evaluate anti-PRM effects. After the in vitro feeding, we collected the blood-fed PRMs and assessed the acaricidal potential. We monitored PRMs that fed on the plasmas and assessed them along the following three criteria: the survival rate (SR), the reproductive capacity (RC), and molting rate (MR). In the first experiment, we used protonymphs and adults to evaluate the effects of plasmas on the SR and RC of blood-fed PRMs. The RC of PRMs that fed on the plasmas from IM2 and IM4 (i.e., chickens immunized with *Dg-Cys*-his) was significantly reduced (Table 1 and Figure 4A), whereas no difference was observed in the SR of PRMs that fed on the plasmas of immunized and control chickens, throughout the observation period (Table 1 and Figure 4B). In the second experiment, we used protonymphs and deutonymphs to assess the effect of the immune plasmas on the MR and SR. While no significant difference was observed in the MR (Table 2 and Figure 5A), the SR of PRMs that fed on the plasmas from IM2 and IM4 was significantly lower than that of PRMs that fed on the control plasma (Table 2 and Figure 5B). These results suggest that the plasma from chickens immunized with *Dg-Cys* increases the mortality of PRMs, especially of protonymphs, and affects their reproductive activity while reducing the generation of progenies.

### 3.5. Enhanced Acaricidal Effect of Dg-Cys-his-Immunized Plasma in Combination with Dg-Ctr1- or Dg-APMAP-Immunized Plasma

**Figure 6:**
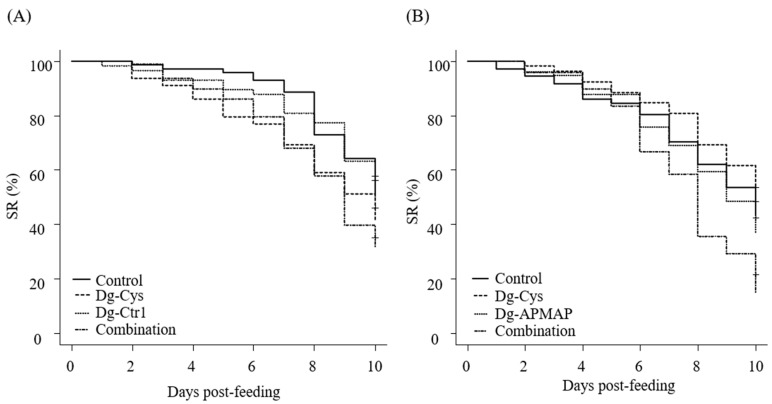
Acaricidal effects of combined immune plasmas on adult mites. The efficacies of “cocktail vaccine” on adult mites were evaluated in vitro. The survival rate (SR) of adult PRMs that were fed with the combined plasmas derived from (**A**) *Dg-Cys*- and Dg-Ctr1-immunized chickens and (**B**) *Dg-Cys*- and Dg-APMAP-immunized plasmas was assessed every day for a ten-day period.

Although plasmas from *Dg-Cys*-his-immunized chickens exhibited anti-PRM properties, their acaricidal effects on adults could not be observed (Table 1 and Figure 4B). Recently, we characterized two more candidate vaccine antigens: copper transporter 1-like molecule (Dg-Ctr1) and adipocyte plasma membrane-associated protein-like molecule (Dg-APMAP). Moreover, acaricidal effects of immune plasmas containing antibodies against these antigens were higher in nymphs than in adults [4,5]. In ticks, several studies have suggested that the “cocktail vaccine”, which combines different vaccine antigens, could potentially have enhanced efficacy than single-antigen vaccines [24,25]. Therefore, to assess the potential of *Dg-Cys* as a cocktail vaccine antigen, we next examined whether the combined use of immune plasmas could enhance the acaricidal effects. We combined the plasma from *Dg-Cys*-his-immunized chickens with other immune plasmas and assessed their acaricidal effects on adult PRMs. The chickens were immunized with the recombinant proteins of Dg-Ctr-1 or Dg-APMAP and exhibited the acaricidal effects against PRMs^4,5^. Upon using immune plasma against a single antigen, the immune plasma of *Dg-Cys* exhibited significant reduction in SR at days five and seven post-feeding compared to the control plasma. Conversely, no acaricidal effect was observed in PRMs that fed on Dg-Ctr1-immune plasma (Table 3 and Figure 6A). Notably, PRMs that fed on the combined plasmas, which contained antibodies specific to *Dg-Cys* and Dg-Ctr1, showed significant decrease in the SR at seven and nine days post-feeding compared to those that fed on control plasmas. In addition, at ten days post-feeding, the SR of PRMs that fed on mixed plasmas was lower than those of PRMs in the immune plasmas against a single antigen (*Dg-Cys*: 10% lower; Dg-Ctr1: 18% lower); however, we did not observe a significant difference (Table 3 and Figure 6A). During the assessment of the combination of *Dg-Cys* with Dg-APMAP, the use of immune plasma against a single antigen exerted no acaricidal effects. Conversely, the combined use of immune plasmas exhibited a drastic decline in the SR from eight days post-feeding compared to those of PRMs fed *Dg-Cys*-immune plasma. Furthermore, the SR of PRMs that fed on mixed plasmas were significantly lower than those of all the other groups at day ten post-feeding (Table 4 and Figure 6B). These data suggest that *Dg-Cys* may function as a cocktail vaccine antigen.

## 4. Discussion

Previous reports have demonstrated the crucial roles of cystatins in ticks’ biological activities, such as blood digestion, egg development, and modulation of host immunity [26,27]. Therefore, the inhibition of these molecules could be an effective control strategy against ticks. However, little is known about the contribution of cystatins to the physiology of PRMs. In the present study, we characterized a newly identified cystatin-like molecule, *Dg-Cys*, which showed marked expression in both fed and starved PRMs [19].

Based on the deduced amino acid sequence and the results of the phylogenetic analysis, *Dg-Cys* was predicted to be a secreted cystatin. Indeed, several studies have reported that secreted cystatins in the salivary glands of ticks help blood sucking and facilitate pathogen transmission by suppressing host immune responses, such as the production of inflammatory cytokines [28,29,30]. In contrast, functions like blood digestion and/or egg development have been proposed for cystatins in the midgut [13,31,32,33]. Here, we revealed that *Dg-Cys* is expressed in the midgut and ovaries, but not in the salivary glands of PRMs, suggesting that it plays important roles in blood digestion and embryogenesis. In addition, recombinant *Dg-Cys*-his inhibited the enzymatic activities of cathepsins L and S, which mediate innate immunity and antigen processing [34,35], suggesting that it might be associated with the modulation of host immunity. In contrast, consistent with previous reports of several cystatins [13,15,36], *Dg-Cys* did not interrupt the enzymatic activity of cathepsin B, thus indicating the selectivity and/or specificity of *Dg-Cys* against its target cysteine proteases. In addition, it implies the existence of other cystatin-like molecules in PRMs. Moreover, in phylogenetic analysis of *Dg-Cys* constituted a discrete cluster from cystatins that are previously reported, including those from ticks, suggesting that *Dg-Cys* potentially has a unique function in PRMs. Therefore, further experiments, such as gene-specific, RNA interference-mediated silencing, are required to elucidate the function of *Dg-Cys* in PRM physiology.

To evaluate the potential of *Dg-Cys* as a vaccine antigen, we assessed the SR, RC, and MR of PRMs. While no difference in the SR was observed in the first experiment, the RC of PRMs that fed on the immune plasma was significantly decreased. These data indicate that *Dg-Cys* plays a crucial role in the development and/or hatching of eggs, as previously reported in cystatins in ticks [37,38], and that vaccinating with *Dg-Cys*-his may control the population growth of PRMs. In the second experiment, no significant differences were observed in the MR. Moreover, in contrast to the data observed in the first experiment, the SR of PRMs that fed on immune plasma was significantly reduced. While the majority of PRMs used in the first experiment were adults, only protonymphs and deutonymphs were used in the second experiment. Although the difference in acaricidal effects observed among developmental stages is unclear, it is possible that the expression pattern of *Dg-Cys* may be different at each developmental stage. Therefore, the expression profile of *Dg-Cys* should be investigated through LCM and RT-PCR analyses at each developmental stage in more detail. Furthermore, in our dataset of RNA-seq analysis, several other cystatin-like transcripts were identified, although their expression intensities were lower than that of *Dg-Cys* [19]. Therefore, the reduced function of *Dg-Cys* conferred by the antibodies may be compensated by other cystatin-like molecules present in the midgut of PRMs and thus, the gene expression analyses and the functional characterization of those molecules should be performed. In addition, the SR of PRMs in the second experiment were relatively lower, even in the control groups, compared with those in the first experiment, suggesting that the inhibition of *Dg-Cys* more critically affects the enfeebled PRMs. Alternatively, adult PRMs may be more resistant against an immunological approach. Recently, we identified two other antigen candidates: Dg-Ctr1 and Dg-APMAP. We found that acaricidal effects of immune plasmas against these antigens were stronger against adults than against nymphs [4,5].

In ticks, a growing number of studies has indicated that combining antigens enhances the efficacies of vaccine [24,25,39]. Here, we demonstrated that the combination of plasmas containing antibodies against *Dg-Cys* and Dg-Ctr1 or Dg-APMAP can augment anti-PRM efficacy. It is noteworthy that the acaricidal effects of the use of combined immune plasmas surpassed those of immune plasma against a single antigen, even though the mixed immune plasmas contained only half the quantity of antibodies against each antigen compared to immune plasma against single antigens, suggesting a prominent potential for a cocktail vaccine including *Dg-Cys*. Although the mechanisms by which the use of combined immune plasmas enhanced anti-PRM properties remains unclear, the disruption of the homeostasis at two different points were more critical to PRMs. Importantly, it may contribute to the reduction in the risk of the selection of PRMs with resistance to anti-PRM effects induced by vaccination [39]. To evaluate the efficacies of the cocktail vaccine in detail, further analyses are needed; including elucidation of the minimal antibody titers of each antigen required to elicit acaricidal effects. Moreover, previous studies indicated that an inadequate combination of antigens could impair vaccine efficacy as there may be antigenic competition [39,40,41]. Therefore, the selection of antigens must be carefully considered, and vaccine antigens should be continuously explored.

Thus, our data suggest that vaccination with *Dg-Cys* in combination with other antigens could be an effective strategy to control PRMs in poultry farms. In this study, however, one-shot immunization did not induce sufficient antibody responses, and only half of the chickens immunized with *Dg-Cys* twice could produce specific antibodies. Therefore, considering the practical applications, further investigation on the appropriate dose, routes, and adjuvants is needed to improve the immunogenicity of the *Dg-Cys*-based vaccine and to efficiently induce antibody responses. In addition, PRM-infestation trials using immunized chickens should be conducted to further evaluate vaccine efficacies.

## 5. Conclusions

In the present study, a novel cystatin-like molecule (*Dg-Cys*) was identified and characterized in vitro. *Dg-Cys* mRNA was expressed in the midgut and ovaries of PRMs, regardless of the life stages and feeding states. Notably, PRMs fed on plasmas from chickens immunized with the recombinant *Dg-Cys* exhibited a significant reduction in the survival rates of nymphs and the reproductive capacity. Moreover, the combined use of *Dg-Cys*-immunized plasmas with either plasmas from *D. gallinae* copper transporter 1 (Dg-Ctr1)-immunized chickens or those from *D. gallinae* adipocyte plasma membrane-associated protein (Dg-APMAP) enhanced acaricidal effects of vaccines. Our data suggest that the immunization with cocktail vaccines including *Dg-Cys* could potentially be effective methods to control PRMs in the poultry industry.

## Figures and Tables

**Table 1:** Summary of the anti-PRM property of *Dg-Cys*-his immunization (first experiment).

	Days Post-Feeding							RC
	1	2	3	4	5	6	7	
Immunized (*n* = 255, nymphs: *n* = 38, adults: *n* = 219)								
No. of dead PRMs	5	12	16	21	35	38	45	1.29
SR (%)	98.04	95.29	93.73	91.76	86.27	85.10	82.35	
Control (*n* = 211, nymphs: *n* = 51, adults: *n* = 160)								
No. of dead PRMs	1	4	13	15	23	26	37	2.24
SR (%)	99.53	98.10	93.84	92.89	89.10	87.68	82.46	
*p* value (Fisher’s exact)	0.228	0.126	1	0.729	0.399	0.499	1	2.38 × 10^−5^ *
Odds ratio	4.19	2.55	1.01	1.17	1.30	1.25	1.01	0.57
95% CI (lower limit)	0.46	0.76	0.45	0.56	0.72	0.71	0.61	0.44
95% CI (upper limit)	199.39	11.02	2.36	2.52	2.39	2.22	1.68	0.75

The data are compared by Fisher’s exact test between immunized and control groups. SR, survival rate; RC, reproductive capacity. * *p* < 0.05 was considered statistically significant.

**Table 2:** Summary of the anti-PRM property of *Dg-Cys*-his immunization (second experiment).

	Days Post-Feeding							MR (%)
	1	2	3	4	5	6	7	
Immunized (*n* = 189, all nymphs)								48.68
No. of dead PRMs	5	6	10	13	34	57	74	
SR (%)	97.35	96.83	94.71	93.12	82.01	69.84	60.85	
Control (*n* = 150, all nymphs)								49.33
No. of dead PRMs	1	3	4	4	14	28	38	
SR (%)	99.33	98.00	97.33	97.33	90.67	81.33	74.67	
*p* value	0.233	0.736	0.280	0.085	0.028 *	0.017 *	0.008 *	1
Odds ratio	4.04	1.60	2.04	2.69	2.13	1.88	1.89	1.01
95% CI (lower limit)	0.44	0.34	0.57	0.81	1.06	1.09	1.16	0.68
95% CI (upper limit)	192.59	10.08	9.07	11.57	4.48	3.28	3.13	1.50

The data are compared by Fisher’s exact test between immunized and control groups. SR, survival rate; MR, molting rate. * *p* < 0.05 was considered statistically significant.

**Table 3:** Summary of the acaricidal effects of combined immune plasmas on adult mites (*Dg-Cys* and Dg-Ctr1).

	Days Post-Feeding									
	1	2	3	4	5	6	7	8	9	10
Control (*n* = 70)										
No. of dead PRMs	0	1	2	2	3	5	8	19	25	34
SR (%)	100	98.57	97.14	97.14	95.71	92.86	88.57	72.86	64.29	51.43
*Dg-Cys* (*n* = 78)										
No. of dead PRMs	0	5	7	11	16	18	24	32	38	46
SR (%)	100	93.59	91.03	85.90	79.49	76.92	69.23	58.97	51.28	41.03
Dg-Ctr1 (*n* = 57)										
No. of dead PRMs	1	2	4	4	6	7	11	13	21	29
SR (%)	98.25	96.49	92.98	92.98	89.47	87.72	80.70	77.19	63.16	49.12
Combination (*n* = 80)										
No. of dead PRMs	0	1	5	8	11	16	25	33	47	54
SR (%)	100.00	98.72	93.59	89.74	85.90	79.49	67.95	57.69	39.74	30.77
*p* value										
Control vs. *Dg-Cys*	1	1	1	0.116	0.019 *	0.068	0.025 *	0.256	0.539	0.970
Control vs. Dg-Ctr1	1	1	1	1	0.891	0.754	0.634	1	1	0.970
Control vs. Combination	1	1	1	0.513	0.254	0.16	0.018 *	0.239	0.019 *	0.074
*Dg-Cys* vs. Dg-Ctr1	1	1	1	1	0.633	0.491	0.496	0.161	0.595	0.970
*Dg-Cys* vs. Combination	1	1	1	1	0.891	0.847	1	1	0.595	0.970
Dg-Ctr1 vs. Combination	1	1	1	1	0.891	0.754	0.468	0.161	0.045 *	0.168

The data are compared by Fisher’s exact test. SR, survival rate. * Holm-adjusted *p* < 0.05 was considered statistically significant.

**Table 4:** Summary of the acaricidal effects of combined immune plasmas on adult mites (*Dg-Cys* and Dg-APMAP).

	Days Post-Feeding									
	1	2	3	4	5	6	7	8	9	10
Control (*n* = 71)										
No. of dead PRMs	2	4	6	10	11	14	21	27	33	40
SR (%)	97.18	94.37	91.55	85.92	84.51	80.28	70.42	61.97	53.52	43.66
*Dg-Cys* (*n* = 52)										
No. of dead PRMs	0	1	2	4	6	8	10	16	20	28
SR (%)	100	98.08	96.15	92.31	88.46	84.62	80.77	69.23	61.54	46.15
Dg-APMAP (*n* = 74)										
No. of dead PRMs	0	3	4	9	9	18	23	30	38	47
SR (%)	100	95.95	94.59	87.84	87.84	75.68	68.92	59.46	48.65	36.49
Combination (*n* = 48)										
No. of dead PRMs	0	2	2	5	8	16	20	31	34	41
SR (%)	100	95.83	95.83	89.58	83.33	66.67	58.33	35.42	29.17	14.58
*p* value										
Control vs. *Dg-Cys*	1	1	1	1	1	1	0.855	1	0.923	1
Control vs. Dg-APMAP	1	1	1	1	1	1	0.859	1	0.923	1
Control vs. Combination	1	1	1	1	1	0.657	0.855	0.026 *	0.070	0.006 **
*Dg-Cys* vs. Dg-APMAP	1	1	1	1	1	1	0.772	1	0.611	1
*Dg-Cys* vs. Combination	1	1	1	1	1	0.357	0.104	0.007 **	0.008 **	0.006 **
Dg-APMAP vs. Combination	1	1	1	1	1	1	0.855	0.062	0.165	0.050 *

The data are compared by Fisher’s exact test. SR, survival rate. Holm-adjusted * *p* < 0.05 and ** *p* < 0.01 were considered statistically significant.

## Data Availability

The data supporting the findings of this study are present within the article and the Supplementary Materials.

## Supplementary Materials

The following are available online at https://www.mdpi.com/article/10.3390/vaccines9121472/s1, Figure S1: The schedule of immunization with *Dg-Cys*-his and sample collection, Figure S2: *Dg-Cys*-his-specific antibody production of immunized chickens, Figure S3: The original uncropped image related to Figure 2, Figure S4:The original uncropped image related to Figure 3, Figure S5:The original uncropped image related to Figure S2, Table S1: Gene expression profiles of a cystatin-like molecule in poultry red mites (PRMs), Table S2: Antibody titers in the plasmas from chickens immunized with *Dg-Cys*-his.
