# Supplementary material for: Characterization of a Novel Cysteine Protease Inhibitor from Poultry Red Mites: Potential Vaccine for Chickens
Source: Vaccines (Basel). 2021 Dec 13;9(12):1472. doi: 10.3390/vaccines9121472 (PMC8706574; doi:10.3390/vaccines9121472)
Supplement: Supplementary file 1 [file vaccines-09-01472-s001.zip › vaccines-1500887-supplementary.pptx]

## Slide 1
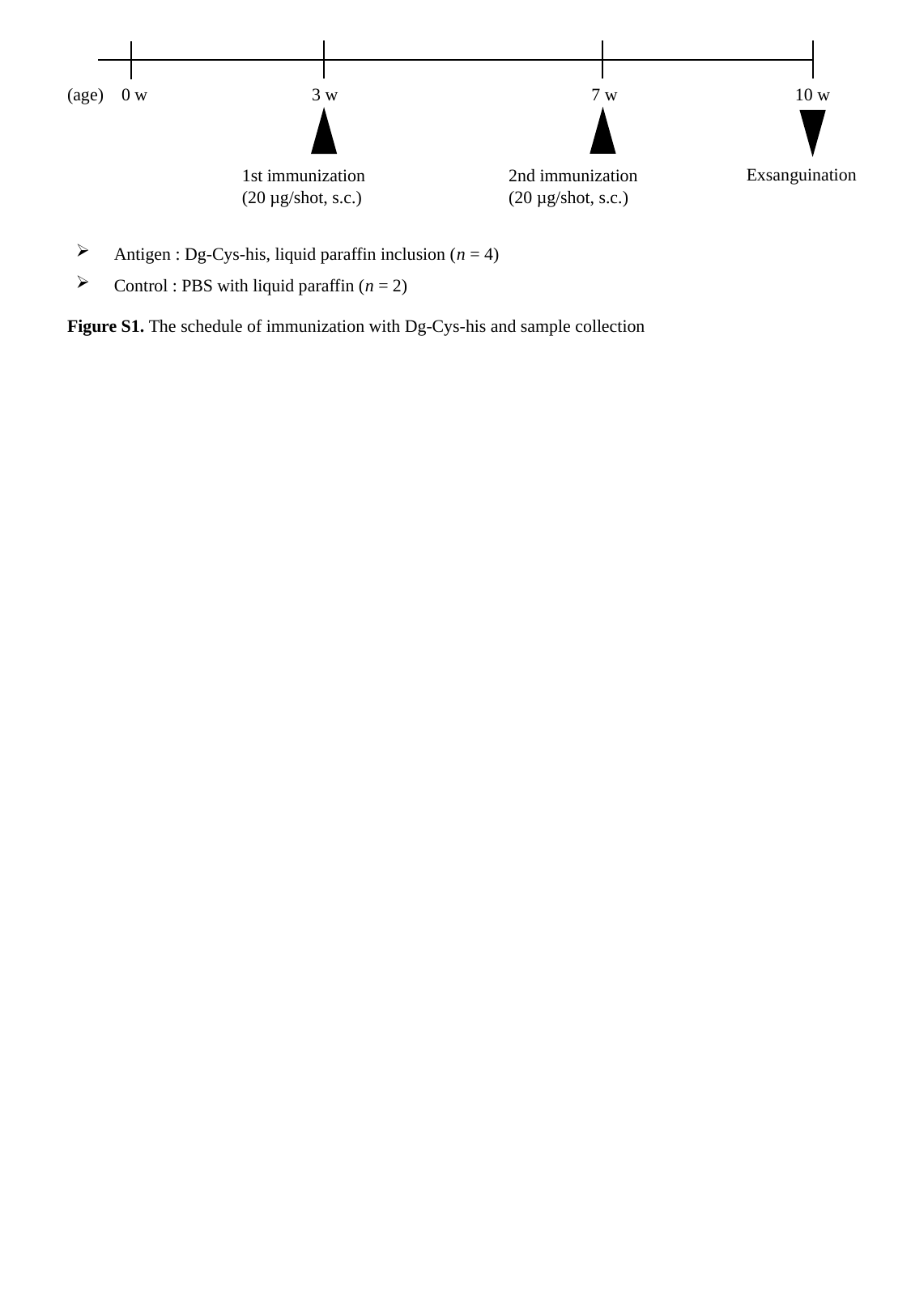

(age) 0 w 3 w 7 w 10 w
Exsanguination
1st immunization
(20 µg/shot, s.c.)
2nd immunization
(20 µg/shot, s.c.)
Antigen : Dg-Cys-his, liquid paraffin inclusion (n = 4)
Control : PBS with liquid paraffin (n = 2)
Figure S1. The schedule of immunization with Dg-Cys-his and sample collection

## Slide 2
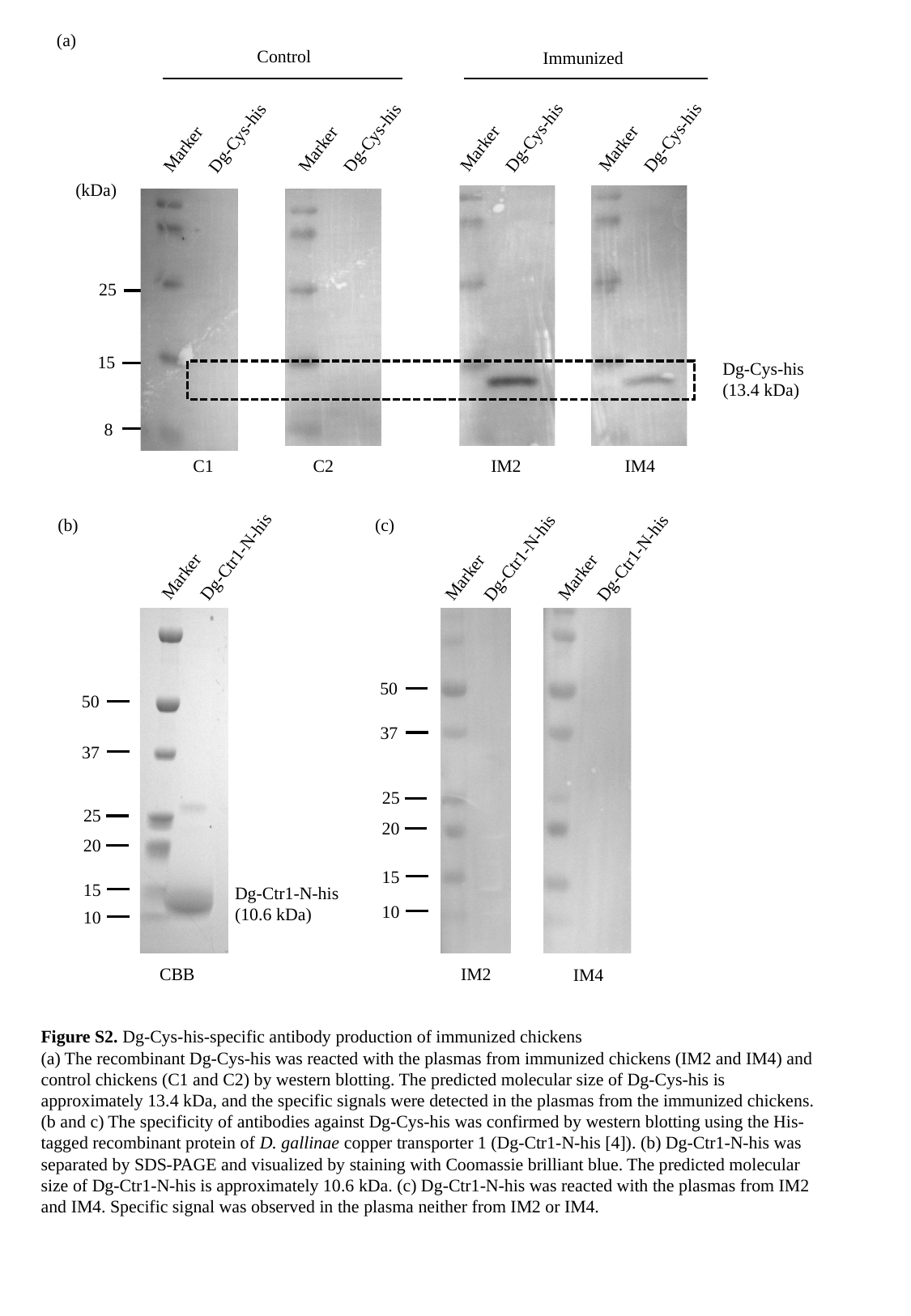

Control
Immunized
Dg-Cys-his
Dg-Cys-his
Dg-Cys-his
Dg-Cys-his
Marker
Marker
Marker
Marker
(kDa)
25
15
Dg-Cys-his (13.4 kDa)
8
C1
C2
IM2
IM4
Figure S2. Dg-Cys-his-specific antibody production of immunized chickens
(a) The recombinant Dg-Cys-his was reacted with the plasmas from immunized chickens (IM2 and IM4) and control chickens (C1 and C2) by western blotting. The predicted molecular size of Dg-Cys-his is approximately 13.4 kDa, and the specific signals were detected in the plasmas from the immunized chickens. (b and c) The specificity of antibodies against Dg-Cys-his was confirmed by western blotting using the His-tagged recombinant protein of D. gallinae copper transporter 1 (Dg-Ctr1-N-his [4]). (b) Dg-Ctr1-N-his was separated by SDS-PAGE and visualized by staining with Coomassie brilliant blue. The predicted molecular size of Dg-Ctr1-N-his is approximately 10.6 kDa. (c) Dg-Ctr1-N-his was reacted with the plasmas from IM2 and IM4. Specific signal was observed in the plasma neither from IM2 or IM4.
(a)
(c)
(b)
Dg-Ctr1-N-his
Dg-Ctr1-N-his
Dg-Ctr1-N-his
Marker
Marker
Marker
50
50
37
37
25
25
20
20
15
15
Dg-Ctr1-N-his (10.6 kDa)
10
10
CBB
IM2
IM4

## Slide 3
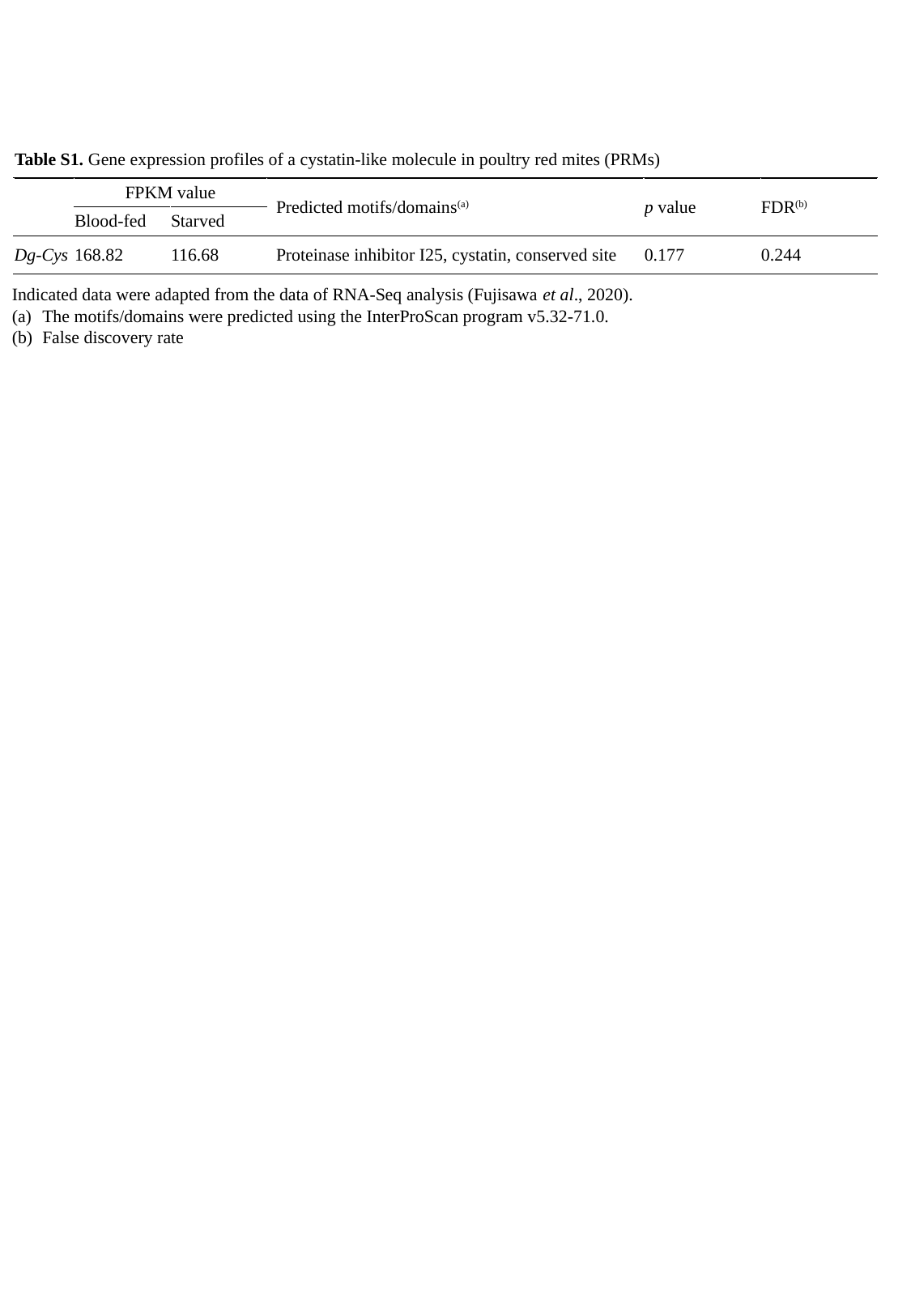

Table S1. Gene expression profiles of a cystatin-like molecule in poultry red mites (PRMs)
| | FPKM value | | Predicted motifs/domains(a) | p value | FDR(b) |
| --- | --- | --- | --- | --- | --- |
| | Blood-fed | Starved | | | |
| Dg-Cys | 168.82 | 116.68 | Proteinase inhibitor I25, cystatin, conserved site | 0.177 | 0.244 |
Indicated data were adapted from the data of RNA-Seq analysis (Fujisawa et al., 2020).
The motifs/domains were predicted using the InterProScan program v5.32-71.0.
False discovery rate

## Slide 4
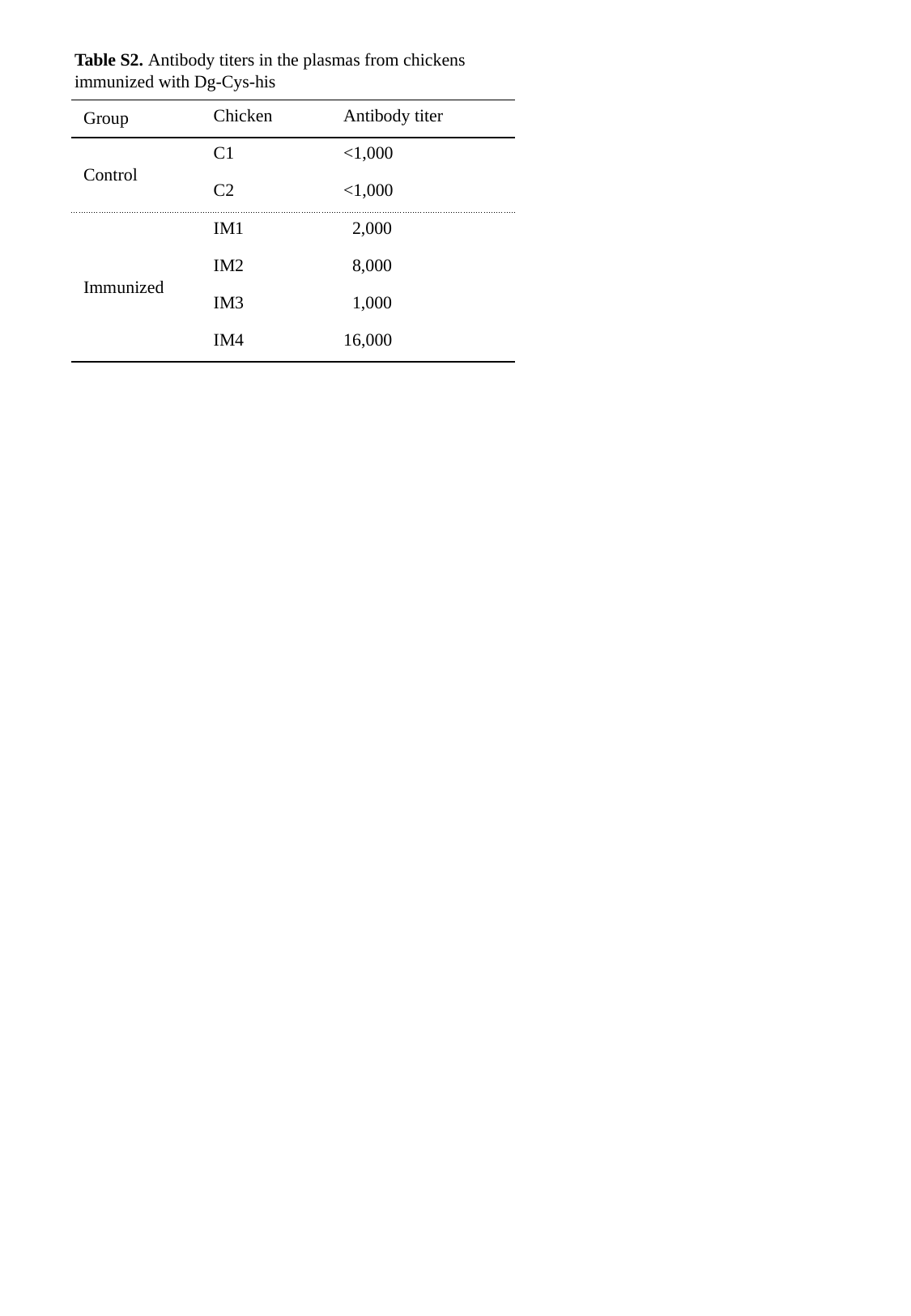

Table S2. Antibody titers in the plasmas from chickens immunized with Dg-Cys-his
| Group | Chicken | Antibody titer |
| --- | --- | --- |
| Control | C1 | <1,000 |
| | C2 | <1,000 |
| Immunized | IM1 | 2,000 |
| | IM2 | 8,000 |
| | IM3 | 1,000 |
| | IM4 | 16,000 |

## Slide 5
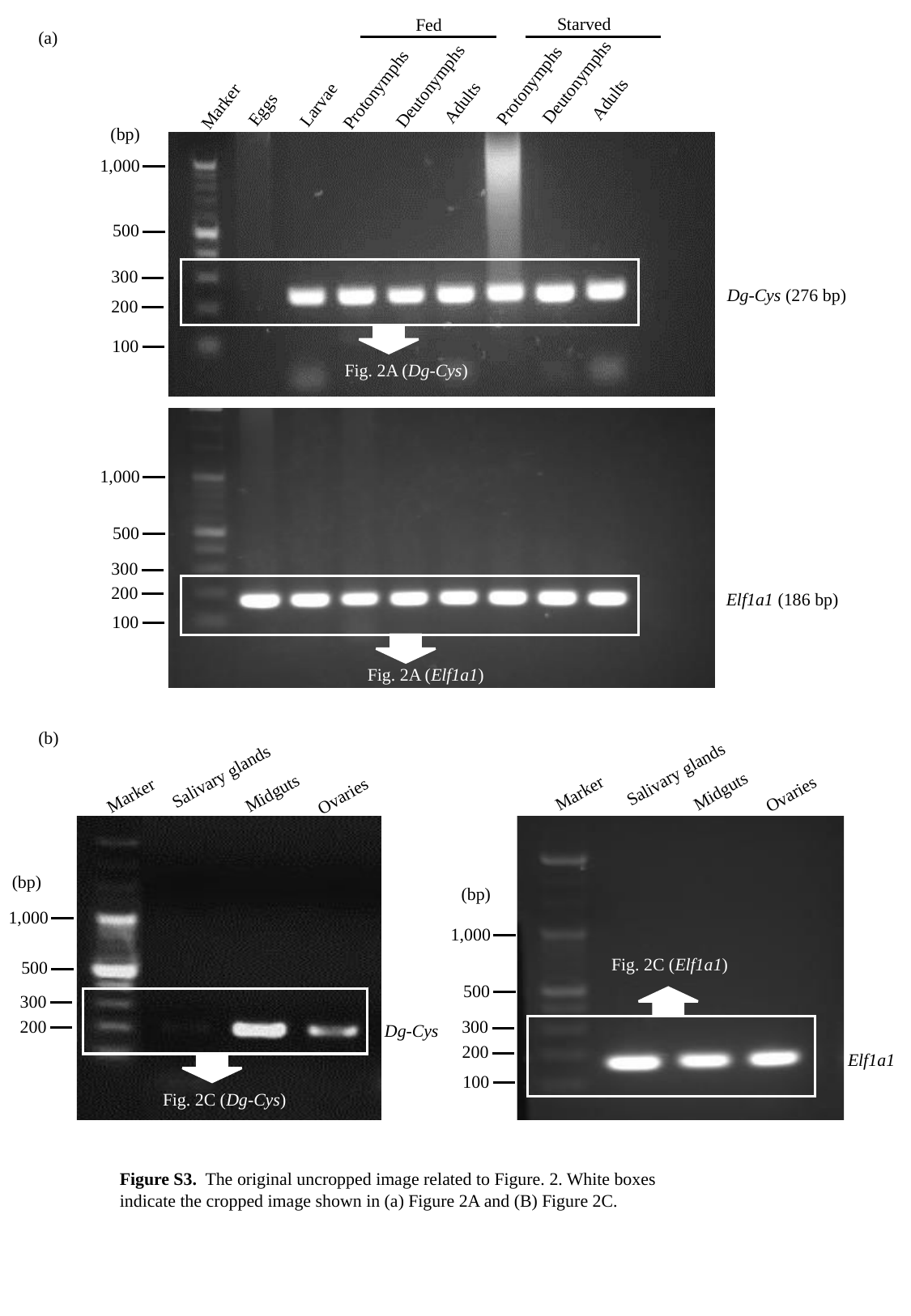

Starved
Fed
(a)
Deutonymphs
Protonymphs
Deutonymphs
Protonymphs
Adults
Marker
Adults
Larvae
Eggs
(bp)
1,000
500
300
Dg-Cys (276 bp)
200
100
Fig. 2A (Dg-Cys)
1,000
500
300
200
Elf1a1 (186 bp)
100
Fig. 2A (Elf1a1)
(b)
Salivary glands
Salivary glands
Marker
Midguts
Marker
Midguts
Ovaries
Ovaries
(bp)
(bp)
1,000
1,000
Fig. 2C (Elf1a1)
500
500
300
200
300
Dg-Cys
200
Elf1a1
100
Fig. 2C (Dg-Cys)
Figure S3. The original uncropped image related to Figure. 2. White boxes indicate the cropped image shown in (a) Figure 2A and (B) Figure 2C.

## Slide 6
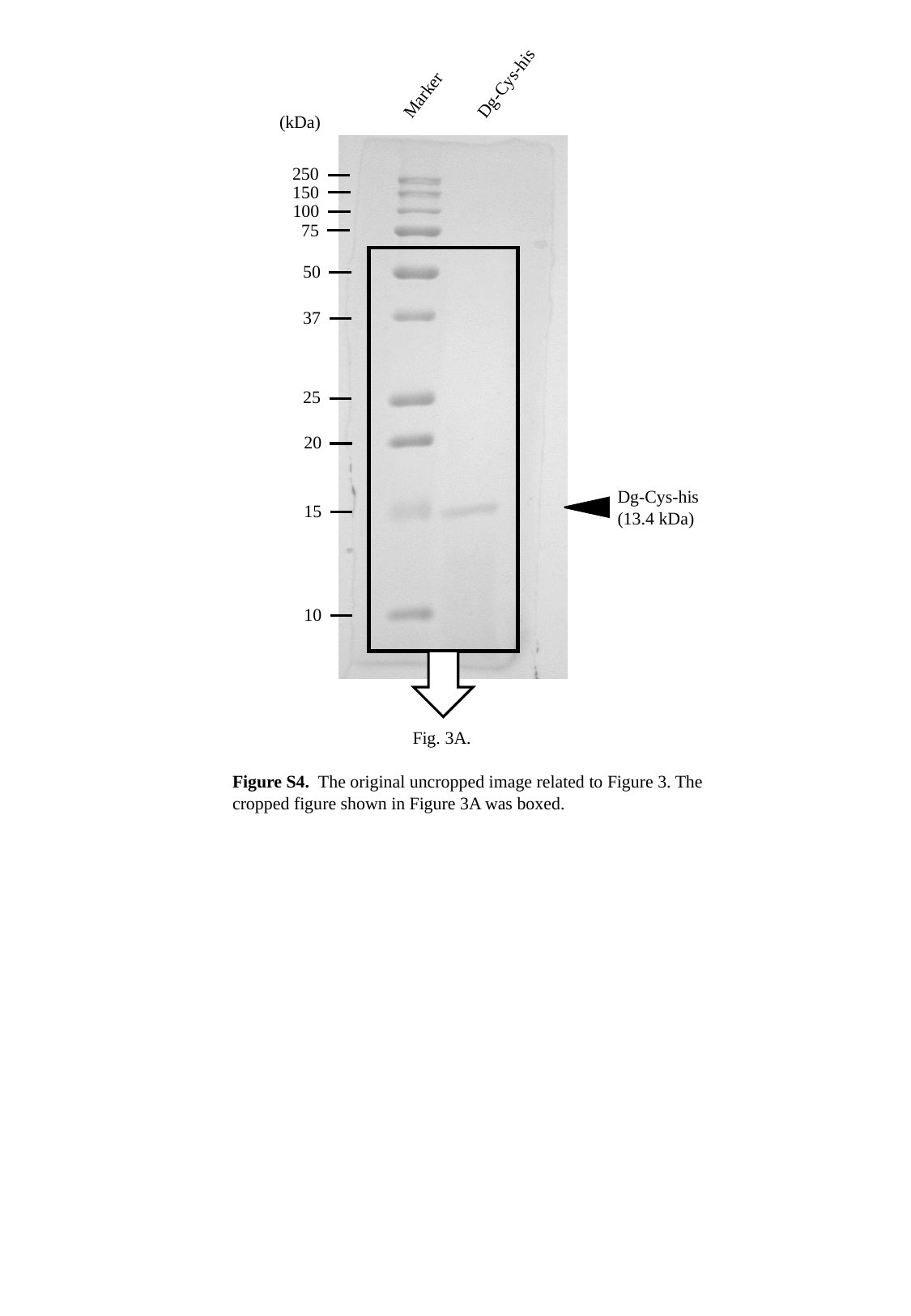

Dg-Cys-his
Marker
(kDa)
250
150
100
75
50
37
25
20
Dg-Cys-his (13.4 kDa)
15
10
Fig. 3A.
Figure S4. The original uncropped image related to Figure 3. The cropped figure shown in Figure 3A was boxed.

## Slide 7
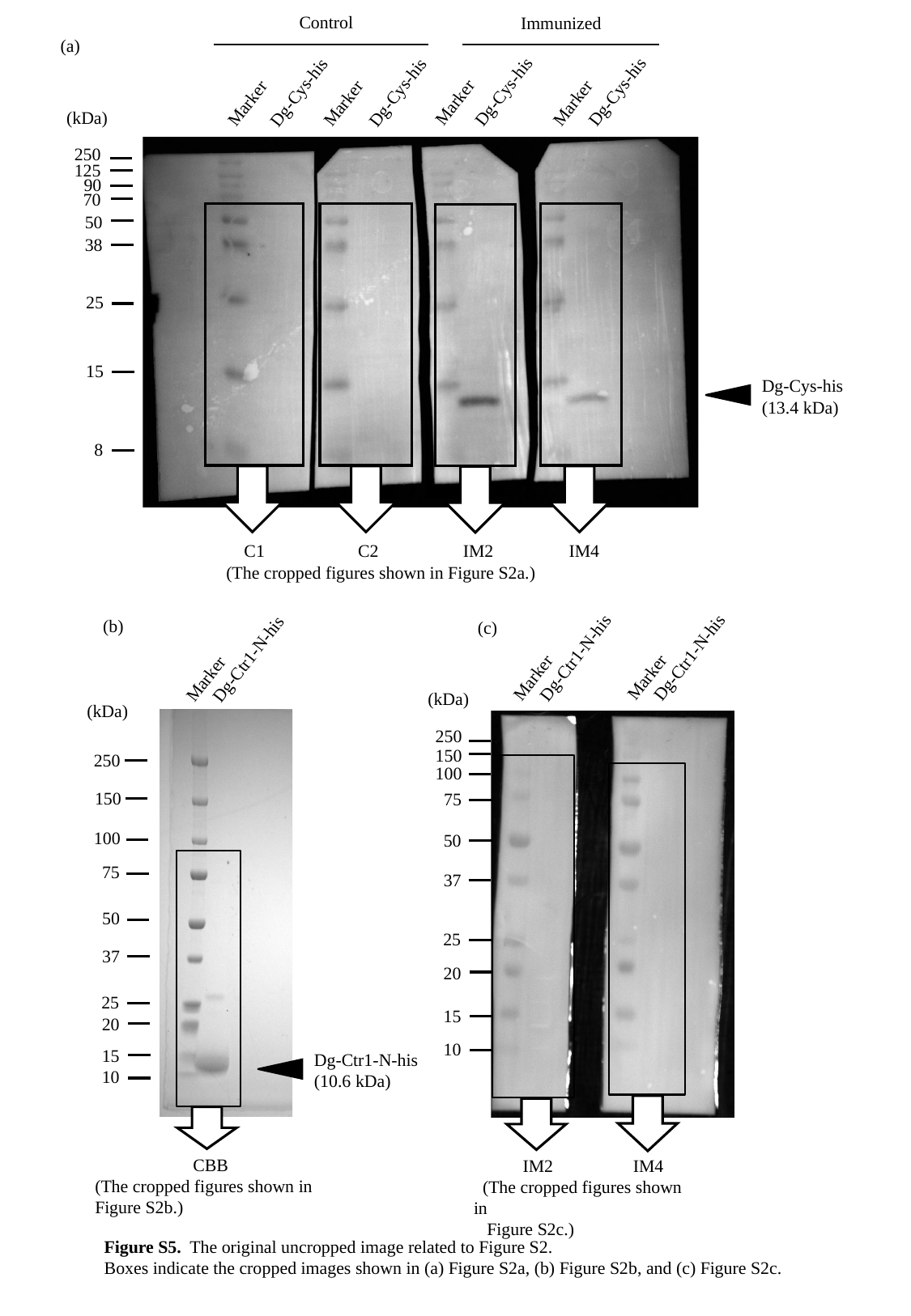

Control
Immunized
(a)
Dg-Cys-his
Dg-Cys-his
Dg-Cys-his
Dg-Cys-his
Marker
Marker
Marker
Marker
(kDa)
250
125
 90
70
50
38
25
15
Dg-Cys-his (13.4 kDa)
8
 C1 C2 IM2 IM4
 (The cropped figures shown in Figure S2a.)
(b)
(c)
Dg-Ctr1-N-his
Dg-Ctr1-N-his
Dg-Ctr1-N-his
Marker
Marker
Marker
(kDa)
(kDa)
250
150
250
100
150
 75
100
50
 75
37
50
25
37
20
25
15
20
10
15
Dg-Ctr1-N-his (10.6 kDa)
10
 CBB
(The cropped figures shown in
Figure S2b.)
 IM2 IM4
 (The cropped figures shown in
 Figure S2c.)
Figure S5. The original uncropped image related to Figure S2.
Boxes indicate the cropped images shown in (a) Figure S2a, (b) Figure S2b, and (c) Figure S2c.
